# DNA-Templated Preparation of Gold Nanoparticles

**DOI:** 10.3390/molecules16108143

**Published:** 2011-09-27

**Authors:** Jeong Sun Sohn, Young Wan Kwon, Jung Il Jin, Byung Wook Jo

**Affiliations:** 1 Department of Chemical and Biochemical Engineering, Chosun University, Gwangju, 501-759, South Korea; 2 Department of Chemistry, Korea University, Seoul, 136-701, South Korea; Email: ywkwon@korea.ac.kr (Y.W.K.); jijin@korea.ac.kr (J.I.J.)

**Keywords:** DNA-Au complexes, DNA-mediated, gold nanoparticles

## Abstract

DNA-mediated gold nanoparticles were prepared by chemical reduction of DNA-Au(III) complex. The DNA-Au(III) was first formed by reacting DNA with HAuCl_4_ at a pH of 5.6. The complex in solution was reacted with hydrazine reducing Au(III) to Au. The reduced Au formed nanodimensional aggregates. The particle distributions were obtained by scanning electron microscopy (SEM) and transmission electron microscopy (TEM). This method resulted in a rather uniform dispersion of Au nanoparticles of near-spherical shape and 45~80 nm in diameter. Gold nanoparticles were embedded and stabilized by DNA.

## 1. Introduction

Deoxyribonucleic acid (DNA) is one of the oldest naturally occurring polymers. Since the discovery of its double helical structure, the science of DNA has been the center of biological science and biotechnology research. The most important molecular characteristic that makes DNA both attractive and successful for designing a wide variety of structures and devices resides in its molecular and submolecular recognition capabilities [1,2,3,4,5]. In particular, DNA has the unique capabilities to build complex nanostructures via self-assembly, which results from hydrogen-bonds formation between base pairs and hydrophilic-hydrophobic interactions. These capabilities can be utilized in the field of the construction of many different nanostructures using DNA as an assembly tool [6]. Moreover, structural modification of natural DNAs may induce new properties and applications that are closely related to nanobioscience and nanobiotechnology of much current interest. One of our recent articles critically reviews the materials science of DNA [4].

Chemical modification of nucleic acid has become an object of attention from the viewpoints of medicinal chemistry as well as material sciences. A nucleobase was synthesized by Tanaka *et al.* [7] for alternative DNA base pairing through palladium complexation. They further developed this concept by directly transforming DNA-bases into palladium-chelating nucleobases [8], but this research was based solely on the synthetic part of DNA and artificial base pairs were limited to the very small numbers found in a DNA-molecule. Aich *et al.* designed a metallic DNA (M-DNA) by incorporating metal into the hydrogen bonds of DNA bases [9] with divalent metal ions such as zinc, cobalt and nickel. It was reported that M-DNA might possess unusual conductive properties and behaves as a molecular wire. The most extensively studied M-DNA compositions are the complexes of DNA with platinum or palladium [10,11] since they revealed potential use as anticancer drugs. The working principle of the bio-templating approach to the preparation of metal nanowires was shown by Braun *et al.* [12] more than a decade ago. They suggested that the small silver aggregates, which are formed on the backbone of DNA, serve as seeds for further cluster growth. Richter *et al.* [13] and Mertig *et al.* [14] studied the formation of well-separated nanoscale size palladium clusters on a DNA template. In these approaches, the sodium ions of DNA backbone are replaced with silver or other metal ions, which is followed by the reduction of the metal ions to metals. The basic concept of DNA metallization matches the success of other approaches for direct metal-growth on biobodies such as viruses and bacteria [15,16,17]. The general scheme for DNA-templated growth of metal structures in three steps was discussed by Richter [18].

In this report we would like to describe an interesting approach to the preparation of gold nanoparticles by chemical reduction of DNA-Au(III) complexes formed by reacting a natural DNA with hydrogen tetrachloroaurate, HAu(III)Cl_4_. The Au(III) ions bind to the bases of DNA, whereas other metal ions such as silver and magnesium ions bind to the phosphate ions [9]. Earlier, we reported the dependence electrical conductance of a DNA-Au(III) complexes on the degree of complexation and also detailed electrical properties of a DNA-Au(III) complex fiber [19]. Complexation of DNA with Au (III) was thoroughly studied in the 1970s by Nandi and coworkers [20,21,22].

## 2. Results and Discussion

**Figure 1:**
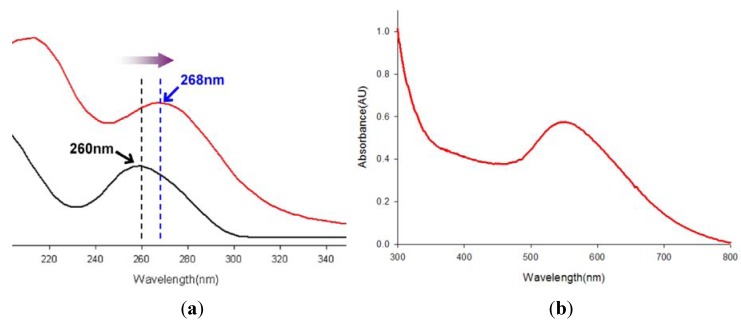
UV-visible spectra of (**a**) DNA (black color) and DNA-Au(III) complex (red color) and (**b**) DNA-mediated gold nanoparticle.

The content of Au in the DNA-Au(III) complex prepared in the present study, which is an intermediate, was found to be 79.80% by weight. We believe that the carbonyl oxygen atoms in guanine and thymine bases and nitrogen atoms in the imidazole rings act as complexing sites [9]. The UV-visible spectrum of the DNA-Au(III) complexes obtained at pH 5.6 after 20 h of reaction is given in Figure 1a. The strong absorption below 230 nm resulted from absorptions of phosphate groups and sugar parts. The second maximum absorption position (λ_max_) of the DNA-Au(III) complex is located at 268 nm. This value is red-shifted when compared with the λ_max_ = 260 nm for DNA which comes from electronic transitions of the aromatic bases. Their absorption edge position is located at about 300 nm.

**Figure 2:**
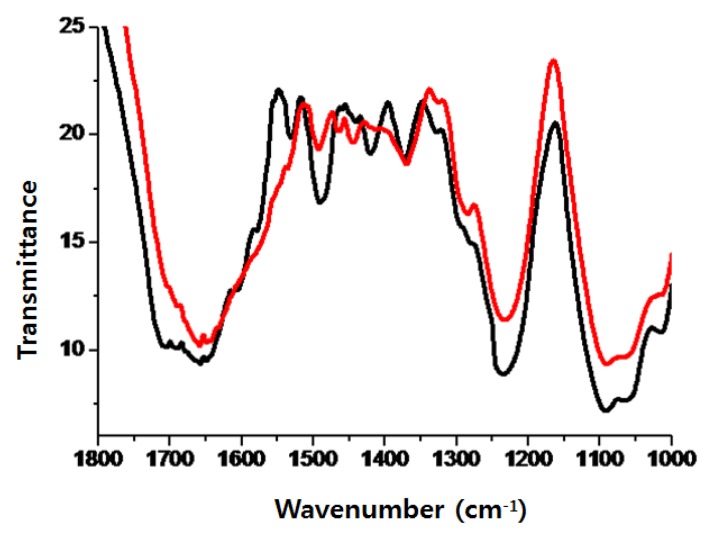
FT-IR spectra of natural DNA (black color) and DNA-Au(III) complex (red color).

Figure 2 compares the FT-IR of DNA and DNA-Au(III) complex. In the 1800~1500 cm^−1^ region, in-plane base vibrations result in absorption bands particularly sensitive to base pairing and base stacking [23,24]. The C6=O6 guanine carbonyl group and C2=O2 thymine carbonyl group are known to be binding sites of metal-DNA [25]. This is reflected in a shift of the carbonyl absorption bands position to the lower side (~1700 cm^−1^ → ~1675 cm^−1^). On the other hand, no band shifts of thymine (C4=O4) (~1655 cm^−1^) and cytosine (C2=O2) (~1647 cm^−1^) carbonyl groups were observed. Absorptions in the 1500~1250 cm^−1^ region are caused by base sugar vibrations. The bands at 1495~1476 cm^−1^ characterize the imidazolic ring vibration and N7C8H bending of adenine and guanine [24]. Its intensity is decreased and a new band is appeared at 1465 cm^−1^, which again indicates that there is a binding between the N7 site of the guanine moiety and Au(III). Sugar-phosphate vibrations appear in the 1250~1000 cm^−1^ region. The antisymmetric PO_2_^−^ stretching band is a characteristic marker for backbone conformation and appears in the A-form at approximately 1240 cm^−1^ [26,27,28]. We cannot observe any special changes in this marker band after complexation with gold. Therefore, it is suggested that the backbone conformation of DNA mostly does not change by the formation of gold complexes and, thus, double strand configuration of gold-DNA complexes is retained.

**Figure 3:**
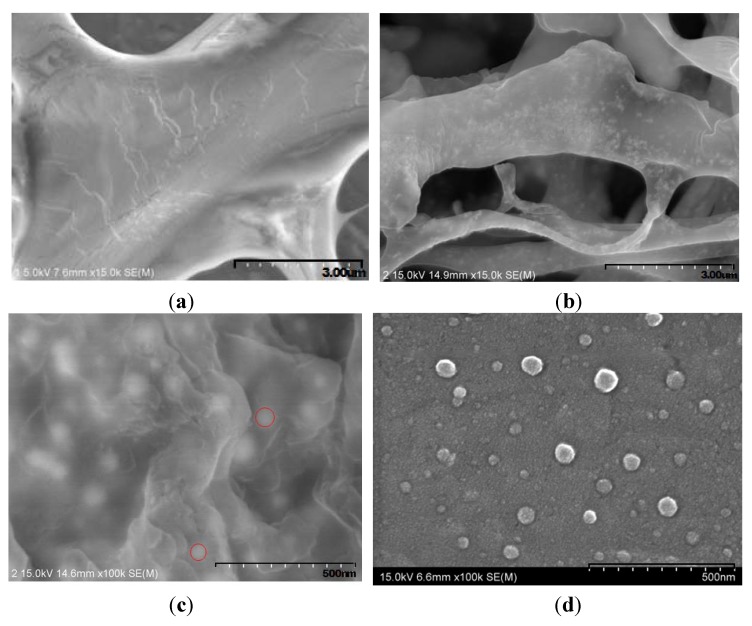
SEM microphotographs of (**a**) DNA (×15.0k), (**b**) low-magnification of the DNA mediated gold nanoparticle (×15.0k), and (**c**, **d**) high-magnification of the DNA mediated gold nanoparticle(×100.0k). (**b**, **c**; prepared from solutions, and d; prepared from washed, precipitated and dried solids).

Figure 1b shows the UV-vis spectrum of gold nanoparticles solution in distilled water. This solution of exhibited a characteristic absorption maximum at 550 nm, which is known to originate from the so-called plasmon absorption by the gold nanoparticles of 45~80 nm in diameter. Figure 3 compares the morphology of DNA (Figure 3a) and DNA containing gold nanoparticles (Figure 3b-d). SEM images show the formation of similarly sized and shaped gold nanoparticles. In Figure 3b-d, Au nanoparticles are observed in the DNA matrix. Figure 3b and 3C are the SEM images of DNA-mediated gold nanoparticle prepared from solutions, and 3d is the one prepared from washed, precipitated and dried solids. As can be seen in Figure 3c, even though the edge of nanoparticles is not well-defined, we can observe nearly spherical nanoparticles with 45~80 nm in diameter.

**Figure 4:**
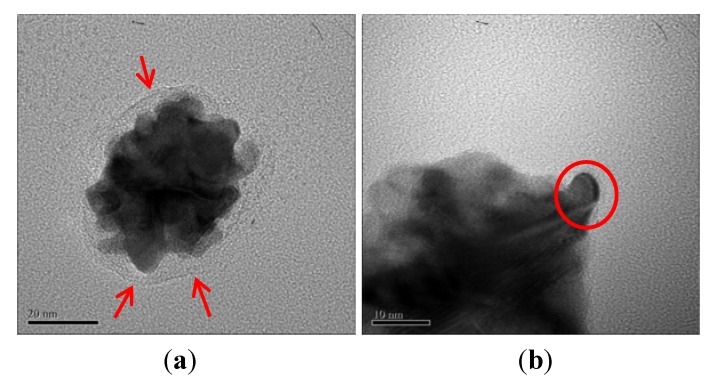
TEM images of the gold nanoparticle with DNA matrix.

TEM images support the formation of gold nanostructures. Interestingly it is observed that DNA-mediated gold nanoparticles (Figure 4a) exhibit clusters in an aggregated form with particles smaller than approximately 10 nm (red circle in Figure 4b). This aggregation leads to clusters with an edge-to-edge distance of roughly 50 nm. This behavior matches the results mentioned above. The nanoparticles also appear to be embedded in a membrane of DNA. Therefore, we can suggest that Au nanoparticles are embedded and stabilized by DNA bases with about 5 nm in thickness (red arrows in Figure 4).

**Figure 5:**
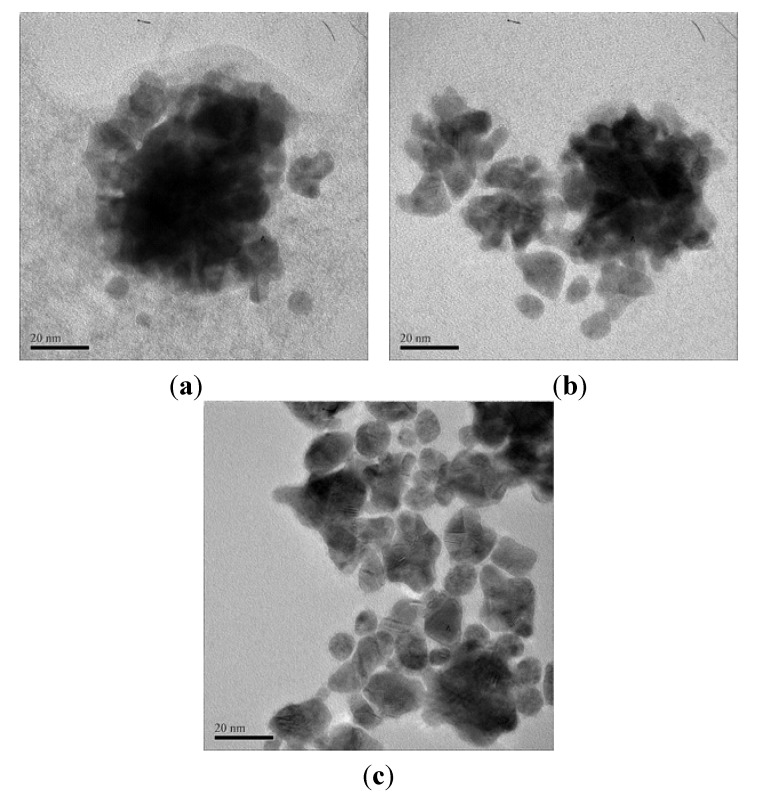
TEM images of gold nanoparticles in DNA matrix with different sonication times: (**a**) 1h; (**b**) 5h; and (**c**) 10 h.

The key issue is how to stabilize Au nanoparticles by DNA bases and to obtain nanoparticles of desired dimensions with narrow size distribution. Thus, DNA-mediated gold nanoparticles were produced by a combination of the rate control for adding reductant and sonication method. Figure 5 shows the TEM images of gold nanoparticles in DNA matrix obtained after different sonication times of 1, 5 and 10 h. When the sonication time increased, aggregated nanoparticles became more disentangled. Figure 5b shows the morphology of a sample after sonication for 5 h. It shows a bimodal size distribution of large agglomerates and small particles. Especially after sonication for 10 h, particles of several nanometers in size were observed.

**Figure 6:**
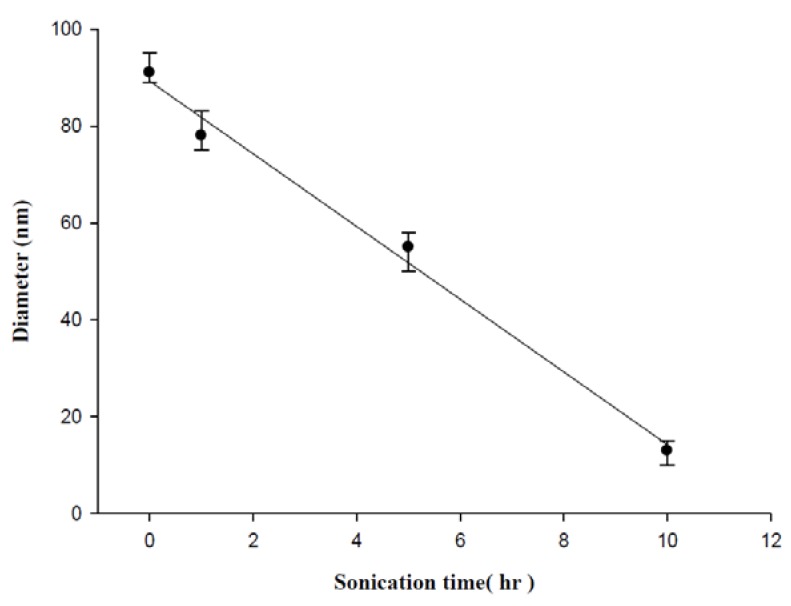
Mean diameter of DNA-mediated gold nanoparticles as a function of the sonication time.

Statistics of the TEM results based on the measurement of at least 100 particles show an almost linear relationship between mean diameter and sonication time. As the sonication time was increased from 1 h to 10 h, the diameter of particles decreased by several nanometers, see Figure 6. The linearity in Figure 6 was not retained after more than 10 h sonication, which is beyond the limits of this study.

## 3. Experimental

### 3.1. Materials

Deoxyribonucleic acid (DNA) and hydrogen tetrachloroaurate(III) trihydrate were purchased from Sigma-Aldrich. DNA (Salmon sperm DNA) used in this study had a molecular weight (MW) of the order of 1.3 million Daltons (approximately 2000 bp). Hydrazine (assay 98%) supplied by TCI was used as the reducing agent. All other chemicals and solvents were obtained from Sigma-Aldrich or TCI and used without further purification.

### 3.2. Synthesis

DNA was dissolved in acetate buffer solution (pH = 5.6) to a final DNA concentration of 1 μM. The solution was swayed slowly at room temperature for 24 h. Hydrogen tetrachloroaurate(Ⅲ) trihydrate (63 mg) was dissolved in H_2_O (6.3 mL) at room temperature. This aqueous stock solution was added to a 1 μM solution of DNA (40 mL). The resulting mixture was incubated for 24 h [19]. Samples of DNA-Au(Ⅲ) complexes for characterization were prepared by precipitating and washing complexes repeatedly with cold ethanol. And then it was dried in vacuum. DNA-templated gold nanoparticle was synthesized by reducing the complexes solution using a solution of hydrazine: 1 M hydrazine solution was added dropwise to diluted gold-DNA solution.

### 3.3. Characterization Methods

FT-IR spectra were recorded on a Nicolet 6700 FT-IR spectrometer as solid KBr pellets. DNA and DNA-Au(III) complexes solutions were dropped on a silicon wafer and dried in the vacuum chamber for XPS measurements. XPS data (Table S1; see Supplementary Material) were obtained using a commercial XPS system (Thermo VG Scientific Escalab 220i-XL) and were fitted by using the commercial XPS software with a sum of 90% Gaussian and 10% Lorentzian functions with Shirley background model. The UV-Vis absorption spectra were collected on an OPTIZEN 2120 spectrophotometer over the 200~800 nm region with a quartz cell. Au contents were determined using inductively coupled plasma atomic emission spectrometry (ICP-AES; OPTIMA 4300 DV, Perkin Elmer).

Scanning electron microscopy (SEM, Hitachi S-4800) was utilized to examine the surface morphology and particle size of gold nanoparticles. Energy dispersive X-ray spectroscopy (EDS, Figure S1; see Supplementary Material) was performed with a Hitachi S-4800 field emission scanning electron microscope (FE-SEM). FE-TEM images of gold nanoparticles were obtained using a Tecnai F20 Transmission Electron Microscope at 200 kV. Samples for TEM analysis were prepared by dropping a solution of a sample onto carbon-film Cu grids (400 mesh, 30 nm). And then they were negatively stained by 2 wt% uranyl acetate solution for 1 minute, soaked with a blotting paper and dried in the air. Sonication was performed at the frequency of 40 kHz (Power Sonic 420).

## 4. Conclusions

We demonstrated that gold nanoparticles could be prepared by chemically reducing DNA-Au(III) complexes. Further studies on the relationship between the size and size distribution of Au-nanoparticles and reaction variables such as reactant concentration, stirring rate, *etc*. are necessary to find optimum conditions for the preparation of Au-particles of desired size.

## Supplementary Materials

Supplementary File 1
