# Supplementary material for: DNA-Templated Preparation of Gold Nanoparticles
Source: Molecules. 2011 Sep 27;16(10):8143–51. doi: 10.3390/molecules16108143 (PMC6264161; doi:10.3390/molecules16108143)
Supplement: Supplementary File 1 [file molecules-16-08143-s001.pdf]

# Supplementary Material

**Table S1.** The electron binding energies of DNA and DNA-Au(III) complexes.

| | N 1s(%) | O 1s(%) | Au 4f7/2(%) |
|---|---|---|---|
| **DNA** | 398.3(17.0) | 530.0(10.7) | |
| | **399.3(25.4)** | 531.0(16.2) | |
| | 400.1(41.9) | **532.1(47.3)** | |
| | 400.9(15.7) | 533.1(18.4) | |
| | | 533.9(7.4) | |
| **Au-DNA complexes** | 398.3(17.0) | 530.0(10.7) | 84.4(22.2) |
| | **399.3(8.8)** | 530.9(14.2) | 85.7(27.0) |
| | 400.1(40.4) | **531.8(30.1)** | 87.9(50.8) |
| | 400.9(17.3) | 532.9(21.9) | |
| | **401.7(16.3)** | 533.8(8.3) | |
| | | **536.5(13.2)** | |
| | | 531.5* (* Data came from Au-OH) | |

**Figure S1.** EDS spectrum of DNA-mediated gold nanoparticle.

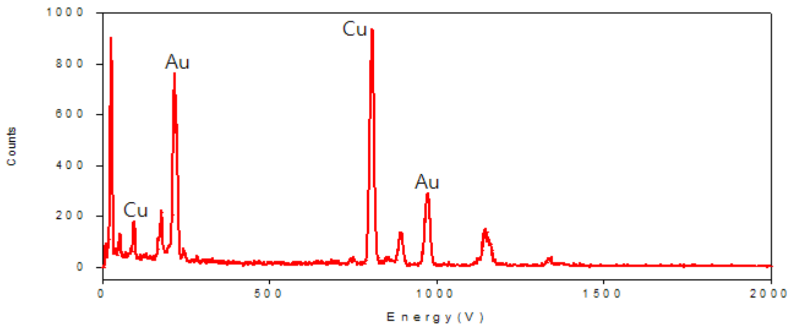


X-ray photoelectron spectroscopy (XPS) allows the analysis of bind sites of the metals with DNA. Table S1 compares the binding energy of N, O, and Au atoms in DNA and DNA-Au(III) complexes. These results confirm that the binding sites of DNA with Au(III) were nitrogen and oxygen atoms in the bases, and three kinds of Au(III) complexes existed in the DNA- Au(III) complexes. As shown in Table S1, we observe appearance of new peaks: one at 401.7 eV for the nitrogen atoms and the other at 536.5 eV for the oxygen atom both complexed with Au(III). The intensity of their original peaks before complexation with Au(III) respectively at 399.3 and 532.1 eV was reduced significantly, much more so with the nitrogen atoms ligands for the complex formation than oxygen atoms. At the same time, the data in the table imply that parts of nitrogen and oxygen atoms remained uncomplexed, although excess. Au(III) was employed. The three Au 4f binding energies of gold-DNA complex indicate that there are three kinds of Au(III) complexes. One (531.5 eV) from the original $AuCl_2(OH_2)$ complex and the other two from DNA-Au(III) complexes. Therefore, we may conclude that there are bindings between DNA and Au(III) with the complex sites being nitrogen and oxygen in the DNA bases. It is well known that a double stranded DNA has the major and minor grooves. The major and

minor grooves open to metal ions to form complexes. The available ligands of the major groove are the carbonyl group of guanine (C6), the nitrogen (N7) of adenine and the carbonyl group (C4) of thymine. And the ligands of the minor groove are the carbonyl groups of thymine(C2) and cytosine(C2), the nitrogens of adenine(N3) and guanine(N3) moieties. These functional groups can act as ligands in the formation of DNA- Au(III) complexes because they have nonbonding electrons.

As shown in Figure S1, Au peaks appear for the DNA-mediated gold nanoparticle at 215 V and 966 V.
